# The Relationship Between Personality Traits, Flow-Experience, and Different Aspects of Practice Behavior of Amateur Vocal Students

**DOI:** 10.3389/fpsyg.2015.01901

**Published:** 2015-12-21

**Authors:** Katharina Heller, Claudia Bullerjahn, Richard von Georgi

**Affiliations:** ^1^Institute of Musicology and Music Education, Justus-Liebig-University GiessenGiessen, Germany; ^2^International Psychoanalytic University BerlinBerlin, Germany

**Keywords:** musical practice, personality traits, flow-experience, singing, motivation

## Abstract

Most of the existing studies on musical practice are concerned with instrumentalists only. Since singers are seldom considered in research, the present study is based on an online-sample of amateur vocal students (*N* = 120; 92 female, 28 male). The study investigated the correlations between personality traits, flow-experience and several aspects of practice characteristics. Personality was represented by the three personality dimensions extraversion, neuroticism and psychoticism, assessed by Eysenck’s Personality Profiler as well as the trait form of the Positive and Negative Affect Schedule. ‘Flow-experience,’ ‘self-congruence’ and ‘fear of losing control over concentration,’ assessed by the Practice Flow Inventory, served as variables for flow-experience. The practice motivation was measured by the Practice Motivation Questionnaire in four categories (‘self,’ ‘group,’ ‘audience,’ ‘teacher’). In addition, the Practice Behavior Questionnaire was used to provide an insight into the practice situation and behavior of singing students. The results show significant correlations: participants with high extraversion-scores experience significantly more flow than less extraverted persons, whereas lesser flow-experience seems to be related to high neuroticism-scores. Nevertheless, there is no influence in flow-experience concerning singing style (‘classical’ or ‘popular’). The longer the practicing time, the more likely students are to achieve flow-experience. However, older singers tend to have less flow-experience. Consequently, singers seem to differ in their personality and practice behavior compared to other musicians. Most of the findings show that having control over one’s instrument is decisive for achieving a performance of high quality, especially for singers. On the other hand, certainty in handling an instrument is essential to arouse a flow-feeling. However, flow-experience seems to be common mainly with amateur singers. In conclusion, this offers a starting point for new research on the psychology of vocalists in greater depth.

## Introduction

Practice behavior has been looked into regarding instrumentalists and with focus on practice strategies, but the personality traits of musicians and their flow-feeling were seldom taken into account. The present investigation, by contrast, was restricted to singers and asked whether their practice behavior differs from the practice behavior of instrumentalists as recorded in existing studies. Especially the correlations with personality traits and flow-experience variables should be studied.

Practicing plays an important part in everyday life of individuals completing a music training, enrolling in music schools, or taking private music lessons. However, especially singers tend to begin their formal training later than instrumentalists (Kopiez, 1998) and are often said to neglect practicing: in a study by Jørgensen (1997) vocal students actually showed the lowest amount of weekly practice time compared with instrumental students. Non-singers even regard a good singing voice as a natural capacity: you have it or you have it not. This might be connected to the fact that the ‘voice’ as musical instrument comprises the whole person: almost all parts of the body have to be coordinated to express ideas and feelings through verbal and musical means. While the respiratory system generates the energy to let the vocal folds vibrate, the air in the vocal tract constitutes the resonator. These body parts must be directed by the brain in order to improve their coordinated functioning and to produce the required pitch, loudness, duration, timbre and so on (Callaghan et al., 2012). Diverse muscles are involved in singing, but, as opposed to the muscles of a drummer’s arm, for instance, one cannot really see them operating. However, to attain the desired performance, they have to be trained as well. Furthermore, learning to sing presents particular difficulties: singers hear themselves differently as compared to their listeners and therefore they must also learn to pay attention to bodily sensations associated with esthetically desirable and physically efficient sounds of their voice. In addition, the voice is an essential element of self-identity: “It helps to define who we are and how other people experience us. It conveys our inner feeling states, both to ourselves and to others” (Welch and Sundberg, 2002, p. 265). Through singing, an individual allows third parties an intimate look at his or hers innermost part, which can make him or her feel naked and strongly vulnerable.

Musical practice also represents one of the most important fields of research in music education and music psychology (cf. for an overview, e.g., Jørgensen and Lehmann, 1997; Gembris et al., 1998; Barry and Hallam, 2002; Lehmann et al., 2007, pp. 61–81; Mahlert, 2007; Jørgensen and Hallam, 2009; Zhukov, 2009; Miksza, 2011; Kaczmaerek, 2012; Lehmann and Jørgensen, 2012), occasionally in conjunction with personality traits (e.g., Harnischmacher, 1993; Miksza, 2006; Butkovic et al., 2015), or flow-experience (e.g., O’Neill, 1999; Roth and Sokolowski, 2011; Marin and Bhattacharya, 2013; Polat, 2013). Nonetheless, most of the existing studies are concerned with instrumentalists only, and singers, as a particular group, are almost neglected (e.g., Ginsborg, 2002 as rare exception). Therefore, the following overview on selected results of research will refer to empirical studies with instrumentalists only. It will also concentrate on the quality of practice and especially the strategies that musicians adopt when practicing.

Most educational and psychological publications on musical practice considered the question of what kind of practice is the most efficient, including historical surveys (e.g., Gellrich, 1992). The late 1920s form the starting point for empirical investigation of musical practice. In the experiment by Brown (1928) a piano score has either to be practiced in a whole without correcting occurring errors, divided into smaller parts that were repeated several times, or played in a whole with correcting occurring errors. Dividing the score into different parts was mainly ineffective but playing it in a whole without correcting any error was highly efficient. Rubin-Rabson (1939, 1940a,b, 1941a,b,c,d) can be seen as the first one to systematically investigate practice behavior of pianists (Reichling, 1989), but the small sample size with nine pianists and the trial design with one and the same sample for related investigations and ignoring the learning-effect are highly questionable. Barry (1992) obtained significantly more improvement on melodic and rhythmic accuracy for 29 brass and woodwind students who practiced with step-by-step instructions and under supervision of an adult in contrast to 26 students practicing freely. Ross (1985) was examining the effectiveness of mental practice, defined as cognitive rehearsal of a piece of music without physically performing on the instrument, in improving the performance of 30 college trombonists. He found out that the combination of mental with physical practice produced the best improvement in performance compared with either mental or physical practice. Likewise, in Coffman’s (1990) study, mental practice alone proved to be inferior to physical practice or physical and mental practice alternated respectively. In his dissertational thesis Kopiez (1990) reached the same conclusion: 108 guitar students of different German universities of music performed better in memorizing a piece of music using physical practice compared with mental practice only or physical and mental practice in combination. Using a sample of students with experience in jazz improvisation and varying the difficulty of a task (performing a tonal pattern according to chord symbols), a high share of physical practice proved to be significantly superior only for the hard task compared with high share of mental practice (Cahn, 2008). Rosenthal et al. (1988) compared 60 college students with brass or woodwind instruments in using different practice strategies. Results showed that listening to a piece of music (‘modeling’) and playing it on one’s own seems to have almost the same effectiveness for mastering a piece of music. However, Henley (2001) could demonstrate that integrating modeling improved rhythmic and tempo mastery but not the pitch performance. The effectiveness of modeling was also supported by the empirical studies of Fortney (1992) and Theiler and Lippman (1995), in which both modeling and mental practice surprisingly appeared to be more effective than free practice in helping to improve students’ instrumental and vocal performing level. Because all these results came from highly controlled experimental situations, Bernardi et al. (2013) chose a more ecological approach: 16 pianists from the University of Music and Drama Hanover were asked to learn two pieces of music, one of them with mental practice and the other with physical practice, on two different days. Each participant was completely free to use any mental practice strategy they desired without any constraints and was allowed to switch between them. After 30 min practicing time and a performance by memory, the mental practice group was free to combine mental strategies with actual piano playing for 10 min. A comparison of performances indicated that mental practice alone showed a much lower level of proficiency than that achieved by physical practice. However, the combination with a relatively short physical practice session revealed almost as good results as only physical practice and therefore was a good preparation for physical practice in restricted time. The inconclusive findings concerning mental practice are presumably due to the varying methodical approaches, settings and aims of the studies with subjects of differing expertise, varied musical instruments, and practice pieces of different style and complexity.

The findings of an observation study by Gruson (1988) already suggested that inexperienced musicians, left to themselves, will tend to play through whole pieces or sections without stopping and do not repeat individual passages that are causing difficulties. But mere repetition may not always be the best strategy for instrumental beginners (Stambaugh, 2011). Sikes (2013) could not even find any significant difference comparing the effectiveness of specific practice strategies on the performance of 40 university string players with non-music majors. Hallam et al. (2012) aimed to explore the development of practice strategies and motivation to practice as expertise develops. A total of 3,325 young musicians at nine levels of expertise, aged 6–19 and playing a wide range of different instruments, were asked to fill in a questionnaire which consisted of a number of statements relating to practice strategies, organization of practice, and motivation to practice. A factor analysis revealed seven factors, of which five proved to be in a statistically significant linear relationship with grade level: “Learners at higher levels of expertise reported adopting more effective practicing strategies and perceived that they were more able to recognize errors. They also ceased to adopt the ineffective strategies of playing through entire pieces, returning to the beginning of a piece if they made a mistake, or correcting errors as they played through a piece” (Hallam et al., 2012, p. 670). More use was made of the metronome and recordings to assist practice and listening to the performance of others with developed expertise. The data did not support a better organization of practice, although this might have been expected. There also was no effect of level of expertise on adoption of analytic strategies and on ease of concentration. Earlier findings of a study by da Costa (1999) indicated that instrumental pupils, in case of a choice between executing a fixed sequence of practice strategies or a freely selected set of strategies from a list of options, will prefer variety and choice. Pupils with more personalized choices also found that their learning process was faster and more effective. Rohwer and Polk (2006) documented a positive correlation between performance improvement and a number of verbalized practice strategies for middle school students. Furthermore, students classified as ‘analytic practicers’ made significantly more gains – even if they did not apply all previous self-reported strategies – than did ‘holistic practicers’ who preferred repeated run-throughs. Moreover, Byo and Cassidy (2008), Christensen (2010), and Oare (2012) could verify a gap between knowing and optimally using these strategies. Wissner (2010) asked 657 instrumental students and autodidacts (aged 8–70, *M* = 22.8) via questionnaire (partly online). Results showed significant differences in practice behavior regarding sex, musical instrument, and auto didacticism. With an increasing degree of self-taught learning, the use of play-alongs, practicing advice in music journals and the daily practicing time surprisingly increases, whereas the use of practicing aids (e.g., metronome) decreases. Autodidacts also were less familiar with practice strategies and did not apply most of them (mere repetition and automated practice as exceptions).

The two empirical studies by Ericsson et al. (1993) are based on a presumed universal theoretical framework applied to the domain of music that explains expert performance as the result of “deliberate practice.” This technical term denotes highly structured, goal-oriented, effortful, and monitored practice that begins in early childhood and is sustained at least 10 years. The studies both confirmed the assumption that the acquisition of musical skills highly depends on a large amount of practicing time (about 10,000 h extended over more than a decade) with highly qualified feedback from a teacher. Both studies dealt with violinists and pianists at a university of music or symphonic orchestras, though. However, the empirical study by Sloboda et al. (1996) with 254 students of music schools aged between eight and 18, fully confirmed the assumption that there is a strong positive relationship between practice and achievement in musical performance. Klöppel (1998, p. 123) legitimately draws the attention to the fact that the success in playing an instrument might also be responsible for the motivation to practice perseveringly. Williamon and Valentine (2000) even saw their findings “in defiance of the argument that quantity of practice is the fundamental determinant of the quality of performance” (Williamon and Valentine, 2000, p. 353). Their investigation of 22 pianists, preparing themselves for a specific performance and classified into four levels of ability, surprisingly could not confirm the prediction that quantity of practice is monotonically related to the musical, communicative and technical quality of performance. In their systematic review of recent research, Varela et al. (2014) additionally claimed that self-regulated practicing, i.e., goal-oriented planning before, during and after musical practice, mostly has a positive but weak relationship with musical attainment. Individuals must be able to control their behavior, environment, and cognitive-affective states autonomously and have to develop an intrinsic motivation and a feeling of self-efficacy. Teachers therefore need to pay greater attention to imparting an enormous amount of practice strategies while increasing awareness in their appropriate use and granting students free choice of repertoire. Further, it is important to provide “specific proximal goals to be accomplished each day” (Duke et al., 2009, p. 311) and not merely information concerning what and how long to practice.

There are only few studies which are concerned with the relationship between musical practice and personality. One of the most fundamental studies probably is the dissertational thesis by Harnischmacher (1993). In three empirical studies included in his thesis, he aimed at exploring instrumental practice as expression of personal behavior. 334 students of eight German universities of music took part in the third study, a survey using a standardized personality test and a new developed inventory for measuring the self-concept of instrumental playing abilities. First of all, the findings showed that music students had differently shaped self-concepts of musical abilities, which were in agreement with the profession they aspired to work in. Therefore, instrumentalists with a more positive self-concept practiced more intensely than, e.g., music educators with a less positive self-concept. However, an upcoming recital seems to have an extrinsically motivating effect for all students. Harnischmacher (1993) could also prove that action-oriented students tended to plan more, possessed a well-structured practice method, used more of their time for practicing and practiced more regularly than state-oriented students. The more recent study by Miksza (2006) investigated the correlation between impulsiveness, locus of control (attributions for success and failure and the perception of their causes) and music practice in a sample of 40 college brass players. He was able to show that less impulsive musicians used their time in a more efficient way, which led to faster success through controlled procedure. In contrast, no significant correlation could be found between locus of control and practice behavior. In a further empirical study by Miksza (2009) impulsiveness, venturesomeness, and mastery-approach motivation proved to be significant predictors of performance achievement of 60 high school wind players. Greenberg et al. (2015) investigated the relationship between personality and musical sophistication in the general population. Results significantly showed, that openness to esthetics was the strongest trait predictor of scores in all musical sophistication domains, even for performance on the musical ability task. Results underline that aspects of musical expertise are linked to Openness to Experience. Müllensiefen et al. (2014) found musical sophistication related to personality traits such as openness to experience and extraversion using the 10-items Personality Inventory as well as Eysenck’s 12-items extraversion scale.

Individual differences in the proneness to experience flow – a state of effortless concentration with absolute absorption in an activity (Csikszentmihalyi, 1995, 2000, 2010; cf. also Csikszentmihalyi and LeFevre, 1989) – may be another potential predictor of practice. Flow can be seen as an optimal state where challenge and skill are in perceived balance. Csikszentmihalyis construct of flow experience provides relevant insight on the significance of musical experiences and on effective practice in music education (Custodero, 2002). It is plausible that flow may lead to longer commitment, since it has been described as a pleasurable state and therefore may also serve as an intrinsic motivator. Parente (2015) clearly states additional benefits flow can arouse while practicing. Known as ‘emergent motivation,’ flow can help to increase the motivation to practice. The motivation to return to an activity arises out of the enjoyment with the experience itself. Each time we experience something good we want to repeat it over and over again. With each experience, individual goals can be realized a little more. Burzik (2007) introduced a holistic, body-oriented practicing method, of which he claimed that it reliably leads musicians into flow-states during their daily practice and helps them to become one with their instrument, sound and musical interpretation. Using the Experience Sampling Method in her study, O’Neill (1999) discovered that high achieving teenage musicians from a specialist music school experienced flow more often than moderate achievers. Sillmann (2008) was able to confirm that jazz musicians experience satisfaction only from playing music, financial aspects are less important. Hechinger (2010) found a positive effect on flow when identifying with practice, which means that individuals who would rather do anything else than practice show lower results in flow. Furthermore, she was able to verify the influence of motivation on flow. Results showed that avoiding mistakes as a form of motivation has a negative correlation with experiencing flow. However, demographic factors such as age and sex did not reveal any significant correlation with flow. In their diary-based study with 37 instrumental pupils and 35 students of music education Roth and Sokolowski (2011) investigated differences between ‘motivational states of control’ with practicing being joyful and flow-driven and ‘volitional states of control’ with no enthusiasm at all and the need to force oneself to practice. They could show that despite of reluctance, the success of practicing was still reaching mean values and practicing therefore was at least rewarding, although the overall practicing time was considerably shorter than when individuals showed keenness to practice. Astonishingly enough, individuals who practiced reluctantly experienced as much flow as those practicing keenly. In a survey on the incentives of practicing according to 44 students of music education and 83 instrumental students at music school level, Roth (2013) could prove that incentives concerning achievement and flow as well as a conjunction of group loyalty and flow are among the most important ones. In this connection music school pupils aged 10–11 and students of music education rated them significantly higher than music school pupils aged 15–16, which might be seen in conjunction with effects of puberty. The first one to be concerned with flow in connection to practicing and personality was Polat (2013). Contrary to her hypothesis, Polat was not able to verify significant correlations between extraversion as a personality factor and flow-experience in a sample of students from a university of music. Looking at 76 piano students, Marin and Bhattacharya (2013) found that flow-experience was significantly related to trait emotional intelligence and daily amount of practice in hours, but neither to the overall duration of instrumental training in years nor to instrumental achievement. Moreover, individual differences among pianists, specific structural and compositional features of musical pieces and related emotional expressions also seem to facilitate flow-experiences. Butkovic et al. (2015) used a Swedish twin cohort of 10,699 individuals in investigating the question of why some individuals practice more than others. They found significant associations with music practice for IQ, intrinsic motivation, music flow, and openness, with music specific flow being the strongest predictor of music practice. Multivariate genetic modeling with openness, music flow and music practice suggested “that the associations between the variables were largely due to shared genetic influences with some additional non-shared environmental influences” (Butkovic et al., 2015, p. 133).

The following hypotheses were formed on the basis of existing theories concerning personality and flow and results of former studies:

H_1a_: If singers have a high extraversion-score, they have higher flow-experience than singers with lower extraversion-scores.
H_1b_: Singers with high neuroticism-scores tend to have less flow-experience because of their anxiety.
H_2a_: Singers who classify themselves as singers of popular music have higher flow-experience than singers of classical repertoire.
H_2b_: The longer the practicing time, the less intense are flow-experiences.
H_2c_: The older singers are, the easier they can experience flow.
H_2d_: Singers who use the Stanislavsky method while practicing have a higher probability to experience flow.
H_3a_: If psychoticism implies more creativity and egocentrism, singers with high psychoticism-scores often practice for themselves.
H_3b_: Singers with high neuroticism-scores practice for the teacher or the audience more often, because in those they can find confirmation.

## Materials and Methods

The study was conducted in full accordance with the Ethical Guidelines of the German Association of Psychologists (DGPs) and the German Association of Psychologists (BDP) as well as the Ethical Principles of Psychologists and Code of Conduct of the American Psychological Association (APA). These guidelines suggest that for the type of research reported here, a formal ethics approval is not necessary. This is due to the fact that the study only made use of completely anonymous online questionnaires and thus, no identifying information was obtained from the participants. Moreover, participants were informed about the aim of the questionnaire, the anonymity of the data, and that participation was voluntary. In accordance with the ethical principles mentioned above, it was not required to obtain written informed consent by the test subjects.

### Sample

The sample comprises a heterogeneous group of German vocal students. Undergraduate as well as graduated students from the Justus-Liebig-University Giessen took part just like a few singers who were not enrolled in a university. Some of the students were registered in musicology or music education study programs. We defined all vocal students as amateur singers, because none of them currently studied or had studied at a university of music. Participants were recruited online through announcement with the help of a university mail distribution list. Moreover, addressees were invited to use the snow ball effect by forwarding the mail. Through this system, the possibility of reaching a large public was maximized. Only subjects taking singing lessons at the moment were allowed to participate in the study. The sample consists of 120 (92 female, 28 male) individuals. Most of the participants were young adults (*M* = 27.37; *SD* = 9.90; range = 14–69 years) of whom 41 classified themselves as singers of popular music and 54 as singers of classical repertoire. The remaining 25 declared themselves as singers of musical theater songs.

### Test Procedure

The survey was carried out via LimeSurvey in German language from January till the end of February 2013. At the beginning, subjects were requested to fill in the entire online questionnaire on their own without leaving any gaps. Due to the programming, there was no possibility to skip any questions. As a result, there was no missing data and each calculation could be carried out for all 120 individuals.

### Psychological Assessments

#### Eysenck Personality Profiler (EPP-D; Häcker and Bulheller, 1997)

The Eysenck Personality Profiler (EPP) is based on the personality-theory of Hans Eysenck (Eysenck and Eysenck, 1987). It includes the scales extraversion vs. introversion, neuroticism vs. stability as well as psychoticism and openness. The test can be carried out as individual or group test with persons aged 14 or older. This personality test consists of 176 items of which 52 belong to the scale extraversion, 50 to the scale emotionality, 61 to the scale risk tendency, and 13 to the scale openness. These three dimensions are all subdivided in four or five subscales. Each subscale consists of 10–14 items, each item can be answered with ‘yes,’ ‘no,’ or ‘I don’t know.’ The internal α-reliability could be proved for each scale with α = 0.90 for neuroticism, α = 0.87 for extraversion, α = 0.79 for psychoticism and α = 0.73 for openness. In general all subscales showed Cronbach’s alpha coefficient between α = 0.70 and α = 0.87 (cf. von Georgi, 2002).

#### Positive and Negative Affect Schedule (PANAS-d-trait form; Krohne et al., 1996)

The Positive and Negative Affect Schedule (PANAS) consists of 20 items, 10 items for each variable (positive affectivity vs. negative affectivity). The schedule aims at statistically recording the situational and/or general mental state of individuals (Crawford and Henry, 2004). The state measure (affect) is determined by the initial question ‘how do you feel at this moment?’ Being in a single situational state of positive affect (PA) literally means being highly concentrated and full of energy. However, low single PA is dominated by the absence of these feelings. In fact, mood states such as sadness and lethargy characterize low single PA. In contrast to PA, high single negative affect (NA) describes subjective distress. This dimension gives an exhaustive account of mood states such as anger, fear, nervousness, disgust, or guilt. Comparatively, low single NA stands for calmness and cheerfulness (Watson et al., 1988). Watson et al. (1988) reported Cronbach’s alpha coefficient for PA with α = 0.88 and NA with α = 0.87. Affectivity as a trait variable is measured by the question ‘how do you feel in general?’ and is strongly correlated with Extraversion (PA) and Neuroticism (NA; Watson, 2000).

#### Übeflowinventar (ÜFI)

The Übeflowinventar (ÜFI; Practice Flow Inventory) was developed by von Georgi and Polat (unpublished questionnaire). In total, 70 items were investigated factor-analytically and arranged on the basis of classical test theory in a three-factor solution. At last, 30 items showed a high reliability – 10 items per scale. The scales were named as follows: ‘flow-experience’ (FLER), ‘self-congruence’ (SKON), and ‘fear of losing control over concentration’ (KKVA). The internal α-reliability could be proved for each scale with α = 0.84 for KKVA, α = 0.81 for FLER, and α = 0.77 for SKON (cf. Polat, 2013). First correlation analyses with the NEO-ffi show that FLER is negatively related to agreeableness, SKON is significantly correlated with openness and consciousness, while KKVA is strongly associated with neuroticism and low consciousness (*p* < 0.01; Polat, 2013).

#### Übemotivationsfragebogen (ÜMF)

The Übemotivationsfragebogen (ÜMF; Practice Motivation Questionnaire) formed the output of a former unpublished pilot study by von Georgi (unpublished questionnaire). It consists of 44 items. These items form four scales of motivation: ‘self,’ ‘group,’ ‘audience,’ and ‘teacher.’ Each scale includes 11 items. The measurement uses a five-point Likert scale. The internal consistencies for the scales are presented as follows: α = 0.88 for self, α = 0.94 for group, α = 0.92 for audience, and α = 0.93 for teacher.

#### Übeverhaltensfragebogen (ÜVF)

All other aspects of practice aside from flow and motivation (e.g., practicing time or strategies of practice) were tested by using the exploratory Übeverhaltensfragebogen (ÜVF; Practice Behavior Questionnaire). This tool of the overall questionnaire was provided to collect general exploratory data. In its structure it follows the questionnaires from Harnischmacher (1993) and Wissner (2010). Selected questions were borrowed and reworded regarding singers. Specific questions concerning practice process or singing style were enclosed additionally.

### Statistical Methods

All data were analyzed using the Statistical Packages for the Social Sciences (SPSS, Version 18). Hypotheses were tested by using multivariate analysis of variance and regression analysis. The level of significance was set at α = 0.05.

## Results

### Descriptives of the Sample

#### Music Training and Field of Profession

As can be seen in **Table 1**, the starting age of music training in general as well as the starting age of vocal training differed significantly for women and men. Clearly, men began their music and vocal training later than women. On average, the music training started at the age of nine and the vocal training at the age of 15. This is only a little bit above what Kopiez (1998) reported, who stated that German singing students began their formal vocal education at a university of music at an average age of 13.2 years.

**Table 1:** Starting age concerning music and vocal training in ages.

	*M*	*SD*	*p*	*t*	95% CI	Cohen’s	*d*
**At which age did you start your music training in general?**						
Total sample	9.16	5.76	0.013	-2.509	-5.465; -0.644	0.542
Female	8.45	6.03				
Male	11.50	4.07				
**At which age did you start your vocal training?**						
Total sample	15.15	9.01	0.020	-2.457	-11.487; -1.072	0.53
Female	13.68	0.71				
Male	19.96	2.45				


*M, mean-value; SD, standard deviation; p, empirical significance; t, t-value; CI, confidence interval; d, Cohen’s d (effect size) with: 0.00–0.10 (no effect), 0.20–0.40 (small), 0.50–0.70 (medium), >0.80 (large).*

Considering the field of profession, only 37 of all participants wanted to or already did work in the field of music. Moreover, the greater part (69 subjects) worked or wanted to work in an extra-musical field. A striking fact is that 100 of 120 participants did not earn their livelihood from singing. **Table 2** shows the distribution regarding the field of profession they worked in or aspired to work in. 30.8% of the sample wanted to or already did work in a music profession, whereas 57.7% worked or wanted to work in an extra-musical field. 11.7% of the sample had not decided yet in which field of profession they wanted to work in future.

**Table 2:** Field of profession.

	Frequency	Percentage	χ^2^	*df*	*p*
Work in the field of music	37	30.8	38.15	2	≤ 0.001
Music teacher at public school	18	15.0			
Singing teacher	1	0.8			
Private music teacher	1	0.8			
Teacher of early education	3	2.5			
Classical trained singer	3	2.5			
Popular trained singer	2	1.7			
Work in an extra-musical field	69	57.5			
Undecided	14	11.7			


*χ^2^, chi-square; *df*, degrees of freedom; *p*, empirical significance.*

#### Singing Lessons

On average, all participants asked took singing lessons for 5 years, whereas the average duration of lessons per day was *M* = 53.92 min (*SD* = 20.83) with maximum duration of 120 min and minimum duration of 20 min. In general, interviewees took singing lessons once a week. 80% of the subjects indicated that their singing teachers told them how to practice at home. Moreover, all of those 80% declared to pay attention to that. Out of all trial participants, only six also gave singing lessons themselves, all others just took lessons.

#### General Practice Behavior

Only 46.7% of the sample and therefore 56 out of 120 interviewees indicated the use of any of nine mentioned practice strategies. ‘Mere repetition’ is the most common of all practice strategies and was used by 52 subjects ‘very often’ and ‘often,’ respectively. Moreover, this strategy was the only one everyone at least seems to know. ‘Practice with rotating attention,’ a strategy first mentioned by Mantel (1987/1999), seems to be the most unknown of all strategies. 23 subjects indicated that they did not even know this method. Moreover, this strategy was the most seldom in use with only 15 subjects who used it ‘very often’ and ‘often,’ respectively (see **Table 3**).

**Table 3:** Use of practice strategies: frequency and percentage (*N* = 56).

	Very often (1)	Often (2)	Seldom (3)	Not at all (4)	Don’t even know it (5)
Mental practice	6	22	13	10	5
*M* = 2.75; *SD* = 1.15	4.7%	17.2%	10.2%	7.8%	3.9%
Mere repetition	27	25	4	0	0
*M* = 1.59; *SD* = 0.63	21.1%	19.5%	3.1%	0%	0%
Varying practice	9	25	11	3	8
*M* = 2.57, *SD* = 1.25	7.0%	19.5%	8.6%	2.3%	6.3%
From detail to total	14	30	8	2	2
*M* = 2.07; *SD* = 0.93	10.9%	23.4%	6.3%	1.6%	1.6%
Multiphase practice	12	22	11	7	4
M = 2.45; SD = 1.17	9.4%	17.2%	8.6%	5.5%	3.1%
Practice at a microscopic level	5	17	22	7	5
*M* = 2.82; *SD* = 1.06	3.9%	13.3%	17.2%	5.5%	3.9%
Automated practice	6	27	12	7	4
*M* = 2.57; *SD* = 1.08	4.7%	21.1%	9.4%	5.5%	3.1%
Stanislavsky method	13	17	14	3	9
*M* = 2.61; *SD* = 1.34	10.2%	13.3%	10.9%	2.3%	7.0%
Practice with rotating attention	5	10	11	7	23
*M* = 3.59; *SD* = 1.41	3.9%	7.8%	8.6%	5.5%	18.0%


**M*, mean-value; *SD*, standard deviation.*

Regarding the ‘Stanislavsky method’ (to put oneself into the mood of the lyrics by thinking of personal experiences and feelings), only 30 of the respondents even know this strategy. This is surprising, especially because it describes something which is said to be very common in singing.

**FIGURE 1:**
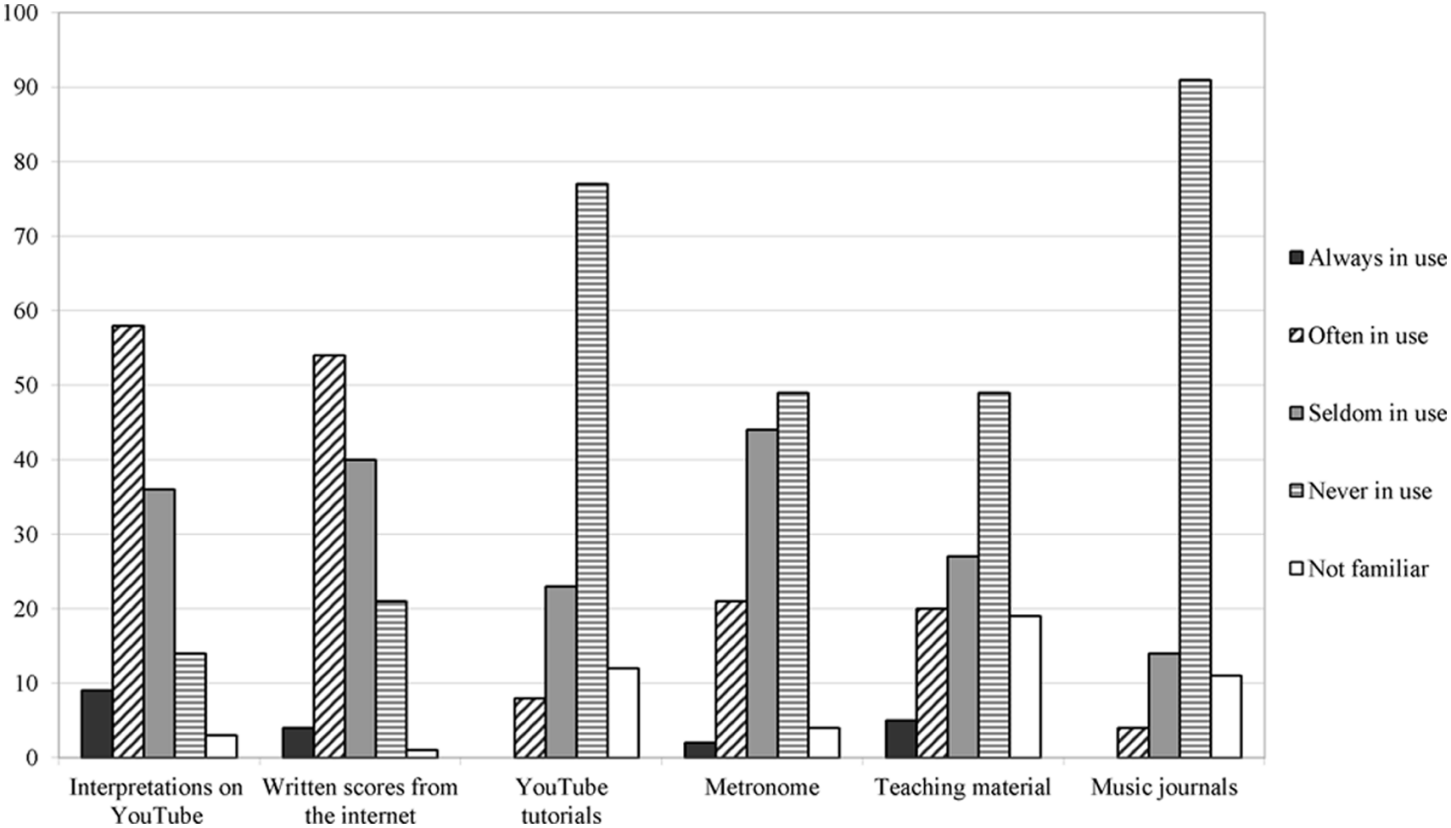
**Use of practice aids**.

Most of the individuals asked practiced once or twice a week. 32 of them did not rehearse regularly. All respondents tended to practice without a break. If there were any breaks, they lasted 9.73 min on average. Mostly, subjects practiced in their own room (73 mentions). Asking them for practice aids, interpretations on YouTube (67 mentions) and written scores from the internet (58 mentions) were the most frequently used. The least used aids are music journals (91 mentions ‘never used’; see **Figure 1**).

Out of all subjects, 25 male and 79 female individuals were singing in a band or an ensemble, only three male and 13 female singers did not sing together with others. It is noticeable that out of all 120, 104 persons sang with others regularly. 56 subjects did only sing in classical ensembles, whereas 48 persons sang in popular music groups but also tended to sing in classical ensembles as well.

### Reliability of the Used Scales

In the present study, these Cronbach’s alpha coefficients were calculated: positive affectivity with α = 0.83, negative affectivity with α = 0.82 (PANAS-d), fear of losing control over concentration with α = 0.81, flow-experience with α = 0.88, and self-congruence with α = 0.82 (ÜFI). According to the practice motivation scales (ÜMF), Cronbach’s alpha coefficients are α = 0.85 for self, α = 0.91 for group, α = 0.88 for audience, and α = 0.83 for teacher. With α = 0.64 for extraversion, α = 0.82 for neuroticism, α = 0.30 for psychoticism, and α = 0.67 for openness, almost all Scales of the EPP show relatively poor coefficients. Given these facts concerning content validity, using EPP seemed to be a risk.

Beyond poor reliability results, another severe problem concerning the EPP became clear very soon: the correlation between measured neuroticism and flow showed an obvious irregularity: there was a significant negative correlation between all neuroticism-subscales and the variable fear of losing control over concentration. This would mean that singers who are more afraid than others tend to have more flow-experiences. From a logical point of view this result seems inconsistent. This confirms the assumption that the measured neuroticism was not independent from other personality dimensions such as extraversion, which would be necessary for further calculation. What is more, a correlation of all neuroticism-subscales with all extraversion-subscales supplied proof of dependence.

To solve this problem, all overlapping results of both scales were isolated and excluded from further calculation using linear regression analysis to compute non-standardized residuals (neuroticism without extraversion).

Because of the problems mentioned above, the main scales of the EPP were not used for further statistical calculations. Instead, the personality dimensions neuroticism and extraversion were tested with the help of the PANAS and the variables NA and PA. According to Watson (2000), PA and NA can be seen as higher order variables of neuroticism and extraversion. This assumption is a consequence of numerous correlations that were found between these variables (Costa and McCrae, 1980; Warr et al., 1983; Emmons and Diener, 1985; Meyer and Shack, 1989; Watson and Clark, 1992).

### Singer’s Practice Behavior in Correlation with Personality Traits and Flow-Experience

A regression analysis revealed a significant positive correlation between the scales extraversion and flow-experience (*R^2^* = 0.042, *p* = 0.025, see **Table 4**). In conclusion, subjects who had higher extraversion scores tend to experience more flow than less extraverted persons. Complementing this finding, results show a significant positive correlation between the scales neuroticism and the fear of losing control over concentration (*R^2^* = 0.087, *p* = 0.001). Apparently, for highly emotionally unstable singers, it is much more difficult to evoke a flow-feeling, because their fear of losing control over concentration is so overwhelming.

**Table 4:** Regression: personality traits (PANAS-d) and flow (ÜFI).

Predictor	Outcome variable	*p*	*R^2^*	*F*	*B*	95% CI
Extraversion (PA)	Flow-experience	0.025	0.042	5.169	0.297	0.038; 0.556
Neuroticism (NA)	Fear of losing control over concentration	0.001	0.087	11.251	0.323	0.132; 0.514


*PANAS-d, Positive and Negative Affect Schedule in German version (trait form); ÜFI, Practice Flow Inventory; PA, positive affectivity; NA, negative affectivity; *p*, empirical significance; *R*^2^, multiple squared regression coefficient; *F, F*-value; *B*, not standardized beta; CI, confidence interval.*

To find out whether the scale flow-experience correlated with the singing style of the participants, a two-factorial MANOVA was statistically calculated. **Table 5** shows that neither singing style and gender respectively alone, nor both variables in combination correlated significantly with any flow-scale. All results were far beyond the significance level *p* ≤ 0.05. Moreover, a division into male and female persons did not deliver any new insights.

**Table 5:** Two-factorial MANOVA: singing style, gender, and flow-experience.

Effect	Wilks’ lambda	*F*	Hypothesis *df*	Failure *df*	*p*	95% CI
Constant term	0.033					
Gender	0.993	0.263^a^	3	112	0.852	24.626; 31.241
Singing style	0.967	0.631^a^	6	224	0.705	24.360; 32.493
Singing style^∗^Gender	0.981	0.366^a^	6	224	0.900	


*^a^Exact statistic. MANOVA, multivariate analysis of variance; singing style, classical, popular vs. musical theater; *F, F*-value; *df*, degrees of freedom; *p*, empirical significance; CI, confidence interval.*

Regarding age and average practicing time, the following results could be found (as shown in **Table 6**): the longer a singer is practicing, the more probable he is to achieve flow (*p* = 0.007). However, it is remarkable that age shows a negative coefficient with β = -0.171. The younger a singer is, the easier he can experience flow (*p* = 0.022).

**Table 6:** Regression: age/average practicing time and flow-experience.

Predictor	Outcome variable	*p*	Total *p*-value	*R^2^*	*F*	*B*	95% CI
Age	Flow-experience	0.022	0.001	0.11	7.266	-0.171	-0.317; -0.025
Average practicing time		0.007				0.066	0.018; 0.114


**p*, empirical significance; *R*^2^, multiple squared regression coefficient; *F, F*-value; *B*, not standardized beta; CI, confidence interval.*

Using stepwise regression, practice strategies and their correlation with flow-scales were explored. A step by step exclusion showed significances for practice with rotating attention (*R^2^* = 145, *p* = 0.004) and the Stanislavsky method (*R^2^* = 0.267, *p* ≤ 0.001; see **Table 7**). The more these methods were used in daily routine, the more flow could be achieved. However, the Stanislavsky method (*R^2^* = 0.243, *p* ≤ 0.001) and varying practice (*R^2^* = 0.340, *p* = 0.007) proved to be suitable for heightening the feeling of self-congruence.

**Table 7:** Stepwise regression: practice strategies and flow-experience.

Predictor	*p*	
**Excluded variables model 1**		***R* = 0.381; *R^2^* = 0.145; *F* = 9.194; *p* = 0.004**
Mental practice	0.076	
Mere repetition	0.590	
Varying practice	0.186	
From detail to total	0.920	
Multiphase practice	0.031	
Practice at a microscopic level	0.160	
Automated practice	0.196	
Stanislavsky method	0.005	
**Included variables model 1**		
Practice with rotating attention	0.004	
**Excluded variables model 2**		***R* = 0.516; *R^2^* = 0.267; *F* = 9.638; *p* ≤ 0.001**
Mental practice	0.175	
Mere repetition	0.747	
Varying practice	0.516	
From detail to total	0.897	
Multiphase practice	0.270	
Practice at a microscopic level	0.511	
Automated practice	0.715	
**Included variables model 2**		
Practice with rotating attention	0.004	
Stanislavsky method	0.005	


**R*, multiple regression coefficient; *R*^2^, multiple squared regression coefficient; *F, F*-value; *p*, empirical significance.*

Regarding personality traits and motivation for practice, two hypotheses were tested. The neuroticism scale clearly showed significant correlations with the motivation scales ‘teacher’ (*R^2^* = 0.044, *p* = 0.022) and ‘audience’ (*R^2^* = 0.064, *p* = 0.005), whereas the psychoticism scale practical-reflected correlates with the scale ‘self’-motivation (*R^2^* = 0.152, *p* = 0.001). There was only one psychoticism subscale used in this case, because of the bad reliabilities of all other subscales (see **Table 8**).

**Table 8:** Regressions: psychoticism/extraversion and practice motivation ‘self’ as well as Neuroticism and practice motivations ‘audience’/‘teacher.’

Predictor	Outcome variable	*p*	Total*p*-value	*R^2^*	*F*	*B*	95% CI
Psychoticism	Practice motivation ‘self’	0.001	0.000	0.152	10.457	0.382	0.161; 0.603
Extraversion (PA)		0.001				0.358	0.144; 0.571
Neuroticism (NA)	Practice motivation ‘audience’	0.005	0.005	0.064	8.035	0.428	0.129; 0.726
	Practice motivation ‘teacher’	0.022	0.022	0.044	5.386	0.308	0.045; 0.570


*PA, Positive affectivity; NA, negative affectivity; *p*, empirical significance; *R*^2^, multiple squared regression coefficient; *F, F*-value; *B*, not standardized beta; CI, confidence interval.*

## Discussion

In general, there is a relationship between positive personality and the ability to get into flow experience. Extraversion was found to correlate positively with flow-experiences and, according to that, flow-experiences correlated negatively with neuroticism scores. Furthermore, socio-demographic markers such as age show a clear relation to flow experience. Amateur singers taking lessons use idiosyncratic practice strategies and seem to differ from instrumentalists in the ways in which they practice. Moreover, practice motivation and personality traits seem to be related.

### Limitations

Principal statistical tests were not carried out separately for men and women with the exception of one hypothesis. Since the participating singers were distributed non-equally regarding sex, tests for gender-specific differences would not be very meaningful. Therefore, the present study did not focus on existing differences in gender. Nonetheless, for singers of popular music a comparison of male with female singers concerning flow-experience (hypothesis H_2a_) might be instructive, especially because most female musicians with skills in popular music are singers (Ebbecke and Lüschper, 1987; Niketta and Volke, 1994; Rosenbrock, 2000). However, neither flow- nor gender-specific differences were statistically significant.

Furthermore, due to a small sample size (*N* = 120), a generalization should be considered with care. In addition, it might be a source of error to infer the actual behavior of the subjects from the individual estimations as stated in the filled-in questionnaire. As declared above, there also might be a difference between knowing a practice strategy and applying it in an ideal way (Byo and Cassidy, 2008; Christensen, 2010; Oare, 2012). Since the study was undertaken online, the subjects were only provided with written instructions which might have resulted in misunderstandings. Personal communication could only take place via E-mail. These problems are well-known. Nevertheless, this method was used to ensure a vast reach which led to a higher response rate at the same time.

Moreover, there is a possibility that the present study on vocal students shows a very special selective sample. Individual aspects such as prosocial behavior that could be common with people who are willing to fill in questionnaires might be the more decisive factor for participating in this study than the fact that they take singing lessons. This could lead to a limitation on the variance of the results.

As a result of the problems mentioned earlier, scales of the EPP were set aside where possible. Nevertheless, psychoticism needed to be questioned by the corresponding subscales of the EPP, but results had to be considered with care. Using the NEO-FFI could possibly have been a reasonable alternative, especially in light of the fact that the EPP was originally used as a survey instrument in the fields of personnel diagnostics. Moreover we were not able to investigate openness as a factor related to practice behavior or flow due to the fact that the EPP was left aside. Considering the results found by Müllensiefen et al. (2014) and Greenberg et al. (2015) it would have been interesting, how openness relates to the variables in our study.

### Test of Hypotheses

The higher the sum value for positive affectivity was, the more flow could be achieved by subjects and therefore, hypothesis 1a is confirmed. Earlier investigations could not find this relation (Polat, 2013). Those different results are probably reasoned by unequal samples. All subjects in Polat’s sample were students of the University of Music Lübeck. They put their main emphasis either on the professional training for playing a musical instrument or on professional vocal training. However, in the present study the vast majority of the subjects consisted of amateur singers who did not necessarily study music but only took singing lessons.

**FIGURE 2:**
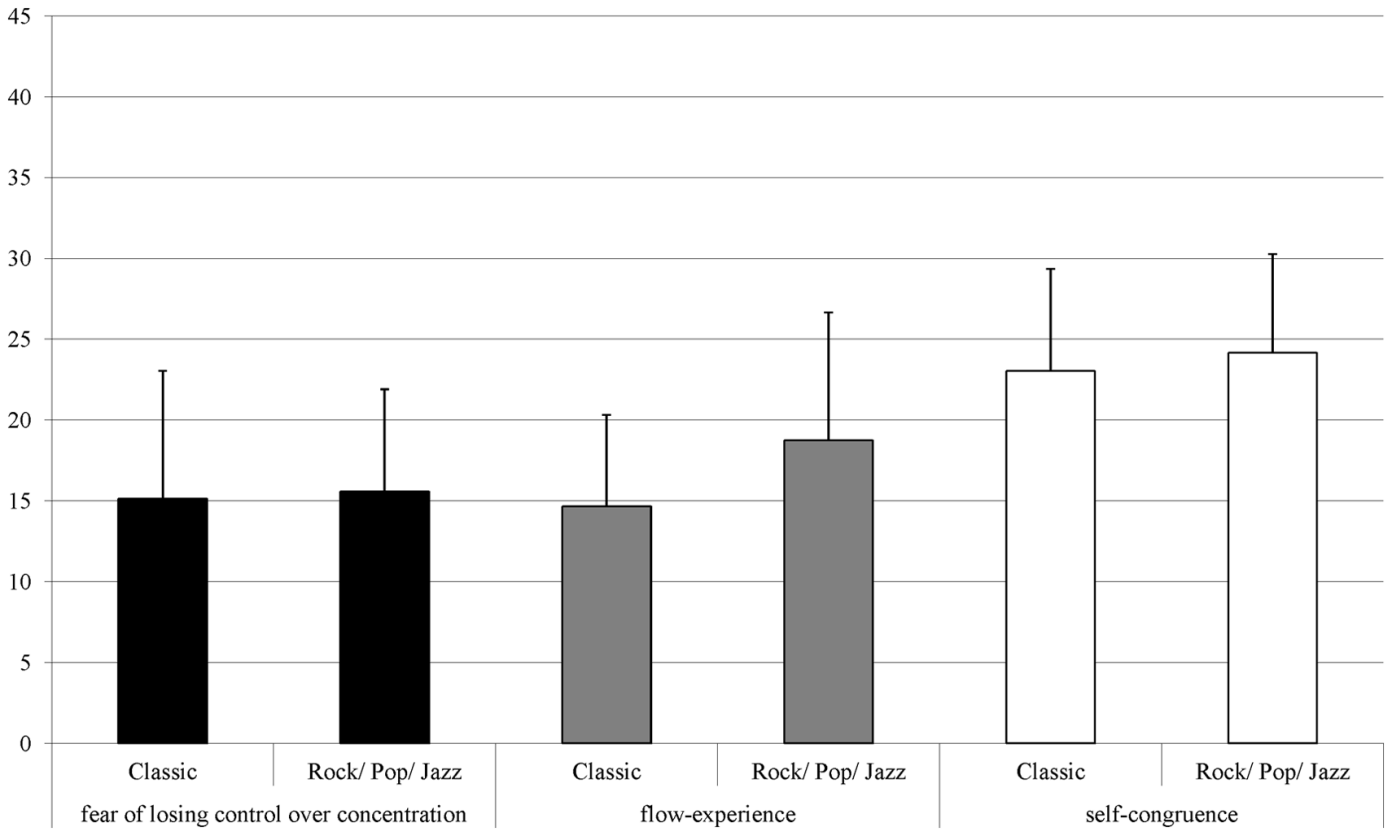
**Statistical averages and standard deviation for the three flow-scales (Polat, 2013, p. 32)**.

**FIGURE 3:**
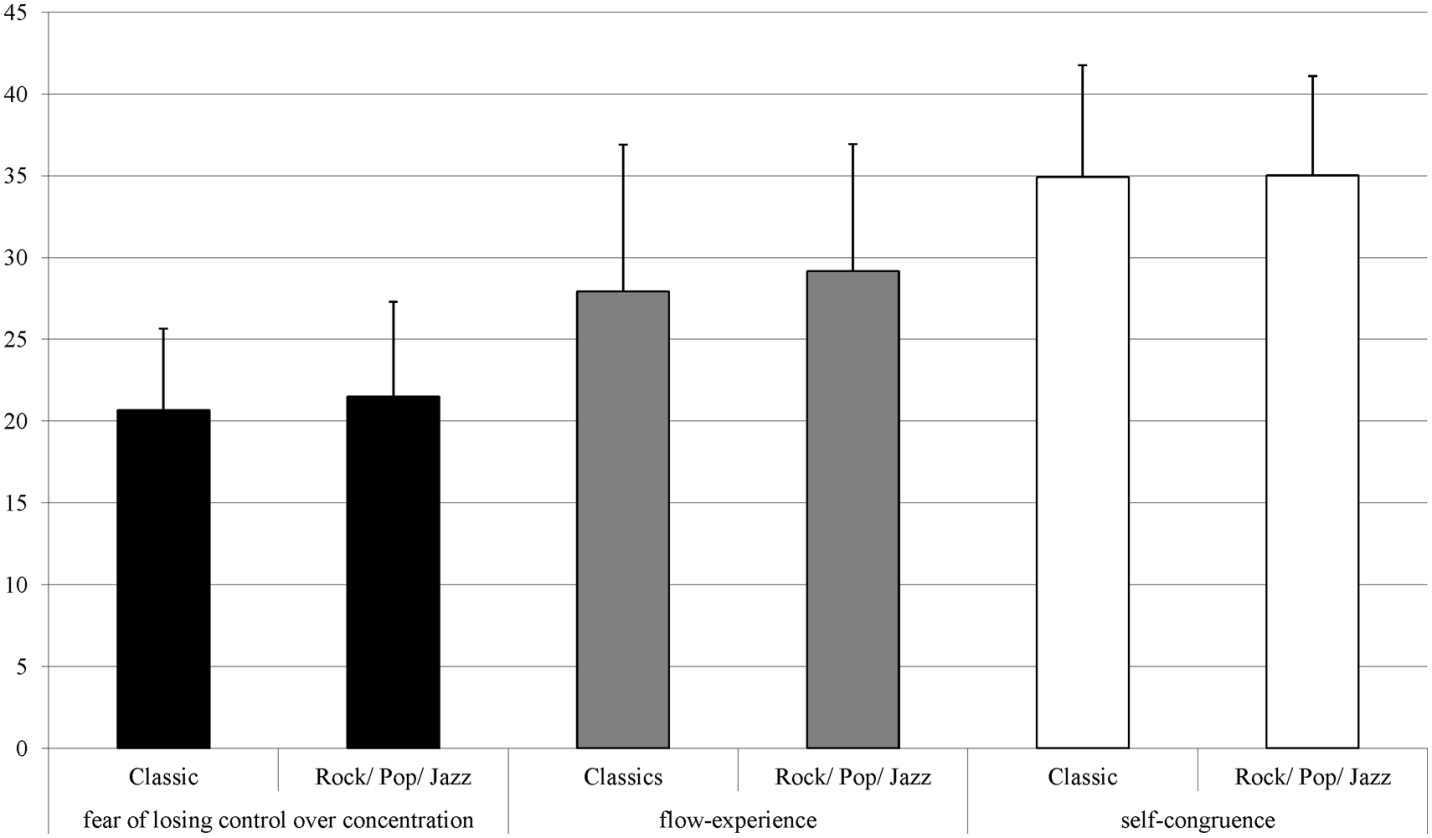
**Statistical averages and standard deviation for the three flow-Scales in the present sample**.

Differences are getting even clearer when considering the statistical average of both samples. **Figure 2** shows the average values of the flow-scales for Polat’s sample. Compared to the present sample shown in **Figure 3**, Polat’s subjects seem to have lower averages in flow. Those differences can be seen as a result of the professional training at a university of music. Really long practicing-times, also in the sense of deliberate practice (Ericsson et al., 1993), and high tension because of performance pressure led to difficulties in experiencing flow, even though students have high extraversion scores. This seems to confirm the assumption that deliberate practice could have a negative impact on arousing flow-feeling.

The next outcome reiterates the aforementioned results in an opposite sense. The higher the neuroticism-scores, the more difficult it is to get into a flow-state. As Csikszentmihalyi already discovered in earlier studies, flow-experience holds a negative relationship with anxiety and concern. By and large, the present study was able to confirm Csikszentmihalyi’s assumptions concerning flow for this very special sample of singers and that is why hypothesis 1b is kept up.

Polat (2013) found a significant correlation between trained music style (‘classical’ or ‘popular’) and flow-experience. Results showed that in comparison to classical musicians, popular musicians have more intense flow-experiences or more flow-experiences in general. Unlike those results, the results of the present study did not show any significant correlation regarding this hypothesis – neither under consideration of all participants, nor when calculations were separated for women and men. In comparison, these results seem to differ because of the different samples. Students at a university of music are probably more often confronted with classical music than amateur singers. Hence, students probably play popular music more often in their leisure time during which they are detached from strict work or any study situation. This allows the students to reduce stress in a practicing situation, which in turn could support flow-feeling. On the contrary, amateur singers do not have such a strict training situation at all. This is why they do not show a significant difference regarding flow and singing style, and because of this, hypothesis 2a has to be rejected. Nonetheless, there are confounding conditions which make some claims questionable. For example, the idea of repertoire or “trained musical style” may be more about the informal and formal ways of teaching and learning than the music itself. Popular music may be treated more like classical music in the context of a voice lesson, whereas a child teaching herself to sing the Queen of the Night Aria by watching YouTube might be more like a garage band experience. Although these examples might be exceptions, the style of teaching and learning should also be controlled in following studies. The results indicate that high expertise is not a necessary precondition for flow, which matches with findings of Marin and Bhattacharya (2013), but contradicts the outcomes of O’Neill (1999). This might have its reasons in different samples. In accordance with Csikszentmihalyi’s Experience Fluctuation Model, flow is more likely to occur when the activity at hand is a higher-than-average challenge and the individual has above-average skills. Either way: challenges and skills should bear an optimal relationship to each other and one must seek greater challenges with increasing skills to achieve or maintain the flow-state (Csikszentmihalyi, 2000, 2010). Csikszentmihalyi presumes that performing a task or any activity over a longer period of time is rather exhausting and tiring. Therefore, it is plausible to assume that practicing time might counteract flow-experience. The present study shows a totally different result: the longer the practicing time, the more likely a singer achieves flow-experiences and this is why hypothesis 2b has to be rejected. The voice as such needs a certain period of time to warm up. To have control over one’s own voice, a singer needs to be warmed up perfectly. To achieve this condition, vocal exercises are necessary before singing a piece of music. Probably singers are not feeling comfortable if their own voice is not warmed up perfectly. Therefore, a longer practicing time helps singers to feel safe in what they are doing, helps them to go beyond themselves and thus leads to more flow.

Earlier observations revealed that, related to several personality traits, flow-experience increases. Age, for example, showed a significant positive correlation with flow, which means the older an individual was the more he or she actually experienced flow (Csikszentmihalyi, 2000; Polat, 2013). On the contrary, Hechinger (2010) could not prove this relation between flow and age in her sample. The present study even found opposite results: the younger a singer was, the easier he or she could achieve flow. Therefore, hypothesis 2c has to be rejected. Those different results probably occur because of the different samples as well as because of the use of different questionnaires. As a person ages, patterns of thought probably become more and more analytical and rational. University of music students as experts probably learned this way of thinking much earlier than amateurs. In Polat’s sample, the musicians did not have to adapt to these thinking patterns because they already knew them (they learned how to use them at the beginning of their professional training). Therefore, for them it is easier to achieve flow when they are older. The present sample has other preconditions. Especially older amateur singers are already adapted through their personal development and practical experiences. Since they learned to cope with analytical and rational patterns of thought a long time before, they are probably not able to experience flow that easily anymore. Because of that, and possibly also because of physical limitations of the singing voice, amateur singers tend to have less flow-experiences with increasing age and more flow-experiences when they are younger.

Educational publications on practice strategies suggest that overall, using more practice strategies increases flow. Especially the Stanislavsky method showed a significant correlation with flow-experience and self-congruence. This is why hypothesis 2d is confirmed. The Stanislavsky method is adopted from acting classes and describes the situation of imagining a special situation to transport feelings in a credible way (Saßmannshaus, 1999; Mantel, 2001, pp. 176–185). Certainly, getting oneself into the emotional spirit of a song helps to really feel a piece of music and to intensely engage with singing, leading to an increased involvement in the activity. This again stimulates flow. In addition, a multi-factorial analysis of variance revealed that the use of practice strategies covariating with flow were independent from personality traits.

Psychoticism scores showed a significant positive correlation with the practice motivation ‘self’ and because of this, hypothesis 3a can be accepted. Nonetheless, these results have to be interpreted with care, because only one psychoticism subscale (practical-reflected) was significant. To practice for one’s own sake, self-reflection is necessary as a logical consequence. Therefore, the findings confirm logical assumptions. Nevertheless, this statement, too, must be considered with care, because the EPP showed unacceptable reliabilities earlier. For the subscales, Cronbach’s alpha coefficient was α = 0.71 and therefore reliable, but bad reliabilities of other psychoticism subscales could still result in the fact that erroneously, there are no significant results shown.

Socially inhibited singers practiced significantly more often for the audience or the teacher than non-reserved singers. Therefore, hypothesis 3b could be confirmed in both its statements. Commensurate with these results a low sense of self-esteem leads to the need of outside validation. Further explorations according to other instrumentalists could be interesting. It can be assumed that this is not a singing-specific phenomenon but can be found in all groups of instrumentalist.

### Future Directions

Results of the present study can give new educational implications, for example regarding flow-experience: practice with rotating attention is the most unknown of all strategies but one of the few that are related with flow-experience. This fact shows that a more frequent use of that strategy while practicing should be encouraged. Moreover, Sillmann (2008) could show that performing music as such is able to incite a satisfactory mood. Hechinger (2010) found out that flow-experience significantly correlates with the activity of practicing. Therefore, it should be recognized that flow has a positive effect on practice. If a person ever achieved a flow-feeling during practice and derived pleasure from it, one might try to re-establish such situations. Also, some sort of positive exhaustion after practice takes place. Practice with rotating attention as a strategy, widely unknown among singers, should therefore be integrated in singing lessons to achieve a more positive practicing situation, which also may increase the efficiency through arousing flow. Concerning teachers who want to create better conditions for evoking flow, Parente (2015) states that teachers in flow have a greater possibility for inducting the same response in their students. To help increasing flow, teachers need to leave room for creativity and openness in their lessons. Moreover, the lesson goals have to be chosen wisely and one has to teach the students as a passionate emerging artist. Of course, this recommendation has to be considered under the restriction that not every singer has a positive connotation with flow. For some individuals the experience of flow might also affect the practicing situation in a negative way (Roth, 2012).

Considering aids used for practice, the latest media clearly show positive correlations with flow (not reported in this article). Using such media while taking singing lessons could possibly help to motivate students for practice and also already stimulate a flow-feeling in singing lessons. Especially watching YouTube-interpretations has positive effects on singers. Nevertheless those interpretations should be chosen carefully and recommended firmly by the teacher to secure a profound basis for learning. This is especially important to avoid problems that may occur later (e.g., learning a completely wrong technique) and that would lead to increased fear again (Wissner, 2015).

For future directions it could be of some importance to carry out a personality test or written questioning with the singing students from time to time. This would make the aims, incentives for learning and current situations of individuals clearer. Lindemann (2002) already took up that idea by having preliminary discussions and protocols when starting to learn an instrument. To create a personal profile, the personal environment as well as musical preferences should be queried. This enables the teacher to guide his student toward meeting his objectives.

In total, recommending general improvements must be considered with care. Research findings cannot validate general statements, because every individual might need a specific concept to achieve the optimal practice situation. Nonetheless, the present results show a valuable approach for improving instructions for singing students in a wide-ranging age class (14–69 years).

Upcoming research should be looking at differences between experts and amateurs, as well as singers and instrumentalists. It would be of great interest to evaluate whether the presented findings are applicable to all singers, all musical amateurs or amateur singers only. Furthermore, it is not quite clear yet whether flow is the precondition for effective practice or whether flow is the consequence of all musical practice regardless of efficiency.

## Conflict of Interest Statement

The authors declare that the research was conducted in the absence of any commercial or financial relationships that could be construed as a potential conflict of interest.
